# Applying a participatory approach to the promotion of a culture of respect during childbirth

**DOI:** 10.1186/s12978-016-0186-0

**Published:** 2016-07-18

**Authors:** Hannah L. Ratcliffe, David Sando, Mary Mwanyika-Sando, Guerino Chalamilla, Ana Langer, Kathleen P. McDonald

**Affiliations:** Women and Health Initiative, Department of Global Health and Population, Harvard T.H Chan School of Public Health, Boston, MA USA; Ariadne Labs at Brigham and Women’s Hospital and the Harvard T.H. Chan School of Public Health, Boston, MA USA; Management and Development for Health, Dar es Salaam, Tanzania; Africa Academy for Public Health, Dar es Salaam, Tanzania; Boston University School of Public Health, Boston, MA USA

**Keywords:** Maternal health, Participatory dissemination, Disrespect and abuse, Respectful maternity care, Tanzania, Health Workers for Change

## Abstract

Disrespect and abuse (D&A) during facility-based childbirth is a topic of growing concern and attention globally. Several recent studies have sought to quantify the prevalence of D&A, however little evidence exists about effective interventions to mitigate disrespect and abuse, and promote respectful maternity care. In an accompanying article, we describe the process of selecting, implementing, and evaluating a package of interventions designed to prevent and reduce disrespect and abuse in a large urban hospital in Tanzania. Though that study was not powered to detect a definitive impact on reducing D&A, the results showed important changes in intermediate outcomes associated with this goal. In this commentary, we describe the factors that enabled this effect, especially the participatory approach we adopted to engage key stakeholders throughout the planning and implementation of the program. Based on our experience and findings, we conclude that a visible, sustained, and participatory intervention process; committed facility leadership; management support; and staff engagement throughout the project contributed to a marked change in the culture of the hospital to one that values and promotes respectful maternity care. For these changes to translate into dignified care during childbirth for all women in a sustainable fashion, institutional commitment to providing the necessary resources and staff will be needed.

## Background

In an accompanying research article, *Mitigating disrespect and abuse during childbirth in Tanzania: an exploratory study of the effects of two facility-based interventions in a large public hospital*, we describe the process of selecting, implementing, and evaluating a package of interventions whose ultimate goal was to prevent and reduce disrespect and abuse (D&A) during childbirth [1]. The study was conducted in an urban regional referral hospital in Dar es Salaam with a catchment area of 1.4 million people. The maternity section of the hospital serves as a referral site to over 40 lower level health facilities, and on average during the study period there were 2060 deliveries, 311 maternal complications, 3 maternal deaths, and 68 neonatal deaths per month. (Facility data) While the study lacked the needed statistical power to definitively measure impact on disrespect and abuse, evidence presented in this commentary suggests that the changes introduced in the facility’s culture have likely decreased the incidence of disrespect and abuse [2]. Here, we describe the evidence and enabling environment that we believe facilitated this change.

## Engaging providers and policymakers to foster collaboration

Generating an open dialogue on disrespect and abuse in a healthcare setting can be challenging. Healthcare administrators, providers, and women seeking care are understandably apprehensive about identifying incidence of D&A within their facility. In an effort to assuage any potential uneasiness towards conducting research around this sensitive area, the study team strategically engaged with key influencers at the facility, district and national levels of the health care system throughout the course of the project. These stakeholders included influential policymakers within the Ministry of Health and Social Welfare who had expressed support for this work in prior conversations with the study team, and leaders of the district-level Council Health Management Team responsible for the facility. We also engaged with key members of the study facility, i.e., nurses and midwives on the frontline of providing antenatal care services and influential members of hospital staff including the matron in-charge, head of obstetrics and gynecology, nurse in-charge of the maternity ward, and the Director of Programs. We posit that the multilevel support generated through our participatory implementation process amplified the effect of the discrete interventions, reinforced the principles of respectful maternity care [3], and had a positive influence on the outcomes observed. Similar to prominent efforts aimed at reducing HIV stigmatization, including programs targeting the prevention of vertical HIV transmission during pregnancy [4], identifying D&A during childbirth at the facility as an issue to be “corrected” and conducting a highly visible project were interventions in and of themselves.

### Sensitization

At the project’s initiation, a series of sensitization meetings were held with policymakers at the Tanzanian Ministry of Health and Social Welfare (MOHSW); the municipal medical officer of health; the Council Health Management Team (CHMT); and hospital administrators and health care providers in the maternity departments of the study hospital. These consultations focused on creating awareness of D&A and generating support to further explore the issue from key maternal health stakeholders at the national, regional and municipal levels. We also consulted with members of the Staha study team, who at this time had initiated a similar study in the Tanga Region of Tanzania [5]. Our ability to engage the MOHSW and increase awareness of D&A was greatly facilitated by this complementary work.

### Participatory dissemination

In the first phase of the project, a baseline assessment found high levels of disrespect and abuse, both as reported by women and as directly observed by trained data collectors [6]. After the baseline assessment was completed, we conducted a three-step participatory dissemination process, the goal of which was to create a culture of transparency, collaboration, and institutional accountability and responsibility. Baseline results were presented initially to the hospital management team and then to providers at the study facility to allow them to internalize and reflect on the findings without assigning blame or attributing fault to specific individuals or areas of the hospital. The second dissemination meeting included hospital providers as well as district-level officials, and focused on receiving input about the feasibility, desirability, and sustainability of potential evidence-based interventions developed from an extensive literature review [7]. After the second consultation, a technical working group—comprised of maternity ward healthcare providers, hospital management representatives, municipal-level health managers, representatives from local and international partner organizations, and study team members—was formed to continue developing interventions that were deemed applicable and appropriate for the study facility. Finally, results were disseminated nationally to obtain feedback and generate high-level consensus on intervention selection. Participants included national representatives from the Ministry of Health; leaders from the regional and municipal health offices; maternal health experts from development partners; academics from medical and nursing institutions; representatives from medical and nursing professional associations; and health care providers and management from the study hospital.

Women who shared their experiences of disrespect and abuse during baseline data collection did not participate in the dissemination process. The decision to limit the audience to those working in the health system was based on the recommendations and requests from key influencers in the region. The rationale was that the initial identification and targeted reduction of D&A needed to be provider-led to allow for acceptability, cooperation, and receptivity of facility-level interventions. Sensitivity around the issue of D&A and tenuous patient-provider relationships—as evidenced by the baseline findings—could have escalated into disagreements and blaming exercises, and negated efforts to mitigate D&A during childbirth.

### Intervention selection

Based on this multi-level participatory process, two interventions were selected: Open Birth Days (OBD) and Respectful Maternity Care Workshops. Open Birth Days included a participatory health education session and tour of the study facility and were open to all women attending antenatal care at the facility during their third trimester. The sessions were designed to improve women’s comfort with the facility and increase birth preparedness by facilitating communication with providers and providing a step-by-step guide to what to expect when they arrive at the facility for delivery. The Respectful Maternity Care Workshop consisted of six modules adapted from the Health Workers for Change curriculum [8]. The goals of the Workshop—which was facilitated by seasoned trainers and respected medical school professors—were for providers to reexamine how their current practice matches their professional codes of conduct, reflect on the needs and preferences of patients, and openly and honestly discuss the barriers that prevent the provision of RMC at the study facility. At the conclusion of the workshop, participants decided upon an action plan that could be carried out of their own accord with minimal influx of external resources. Steps taken by the facility through this action plan included improved use of privacy curtains, changes to staffing structure, and improvements in the speed of overtime payments, among others.

**Fig. 1:**
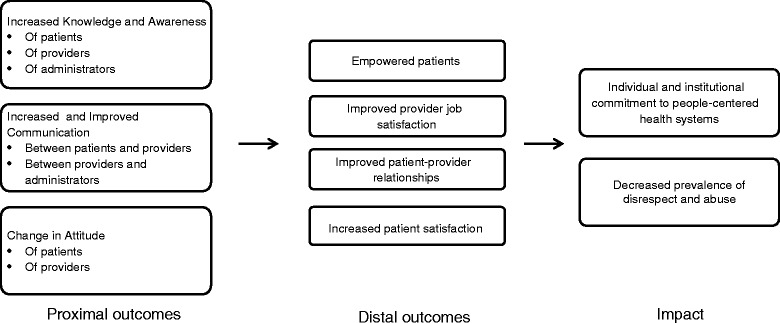
Theory of Change

Rollout of the selected interventions began in April 2014 and continued through October 2014. Reflecting the success of the participatory selection process, nurses in the antenatal ward at the facility began OBD in December 2013 immediately after the national dissemination meeting using their own time, resources, and ideas to drive implementation. Beginning in April, these processes were standardized for implementation and evaluation, and the significant support from facility staff continued throughout the implementation period. The accompanying research article describes in detail the complete implementation and evaluation protocol, and provides evidence from routine monitoring and evaluation activities of marked progress towards achieving proximal and intermediary outcomes targeted under the project’s theory of change (Fig. 1) [1]. In total, 362 women participated in the Open Birth Days Sessions (100 % acceptance rate) and 76 staff from the maternity ward participated in the RMC Workshop (86 % participation rate). Evidence from multiple monitoring tools shows that, over the course of the intervention, there were improvements in both proximal and distal outcomes including: patient knowledge of their rights and birth preparedness, provider knowledge of patient rights, provider attitudes, provider job satisfaction, and patient-provider communications and interactions. Additionally there is evidence that patients were more empowered after participation in an Open Birth Day session, and patient satisfaction with their delivery experience improved substantially from baseline (75.8 % very satisfied compared to 12.9 % at baseline).

## Evidence of a substantial decrease in incidence of disrespect and abuse

In addition to the successful achievement of intermediate outcomes noted in the accompanying research article, all available evidence points towards a declining trend in D&A incidence, both as reported by women and as re- corded by trained observers [1]. Among women interviewed in their homes four to six weeks after delivery at baseline, 70 % reported experiencing any form of disrespect and abuse. At the time of evaluation, 18 % of women interviewed four to six week post-delivery reported experiencing any form of D&A (Table 1). Furthermore, reports of each category of disrespect and abuse also declined dramatically compared to baseline. For example, physical abuse, non-confidential care, lack of privacy, and non-dignified care were reported by more than 50 % of respondents at baseline, compared to less than 5 % after the intervention was concluded.

**Table 1:** Patient reported experiences of disrespect and abuse

Type of Disrespect and Abuse	Baseline Exit Interview *N* = 1914 *n* (%)	Baseline CFU *N* = 64 *n* (%)	Evaluation CFU *N* = 149 *n* (%)	Difference between Evaluation and Baseline CFU %
Any form of disrespect or abuse	278 (15)	50 (70)	26 (18)	−52
Physical abuse	84 (5)	33 (52)	2 (1)	−51
* Kicked*	2 (0.1)	1 (2)	0 (0)	−2
* Pinched*	22 (1)	3 (5)	0 (0)	−5
* Slapped*	23 (1)	17 (27)	1 (1)	−26
* Pushed*	12 (0.6)	4 (6)	0 (0)	−6
* Beaten*	4 (0.2)	5 (8)	0 (0)	−8
* Episiotomy without anesthesia*	1 (0.1)	1 (2)	0 (0)	−2
* Tied to the delivery bed*	2 (0.1)	0 (0)	0 (0)	0
* Other*	17 (0.9)	9 (14)	1 (1)	−13
Non-consented care	4 (0.2)	3 (5)	1 (1)	−4
* Abdominal Palpation*	2 (0.1)	0 (0)	0 (0)	0
* Vaginal Examination*	4 (0.2)	1 (2)	0 (0)	−2
* Episiotomy*	0 (0)	1 (2)	0 (0)	−2
* Other*	0 (0)	1 (2)	1 (1)	−1
Non-confidential care	32 (2)	34 (54)	2 (1)	−53
* Discussed personal issues in earshot of other clients*	1 (0.1)	1 (2)	0 (0)	−2
* Health information discussed with non-health staff*	0 (0)	2 (3)	0 (0)	−3
* Other*	1 (0.1)	0 (0)	1 (1)	1
Lack of Privacy	35 (2)	34 (53)	5 (3)	−50
* Uncovered during delivery or examination*	17 (0.9)	31 (48)	4 (3)	−45
* No screens blocking view during delivery or examination*	16 (0.8)	29 (45)	0 (0)	−45
Non-dignified care	121 (6)	34 (54)	7 (5)	−49
* Shouted at*	35 (2)	24 (38)	0 (0)	−38
* Scolded*	90 (5)	176 (25)	6 (4)	−21
* Threatened to withhold services*	1 (0.1)	1 (2)	0 (0)	−2
* Called by insulting name*	3 (0.2)	0 (0)	0 (0)	0
* Laughed at or scorned*	3 (0.2)	0 (0)	0 (0)	0
* Other*	12 (0.6)	1 (2)	1 (1)	−1
Abandonment of care	147 (8)	33 (52)	21 (14)	−38
* While in Labor*	104 (5)	22 (34)	7 (5)	−29
* While Delivering*	60 (3)	12 (19)	11 (7)	−12
* While experiencing a complication*	1 (0.1)	0 (0)	0 (0)	0
* After delivery*	2 (0.1)	0 (0)	2 (1)	1
* Other*	5 (0.3)	0 (0)	1 (1)	1
Detention in facilities	4 (0.2)	1 (2)	1 (1)	−1

Results from the direct observations of patient-provider interactions also show marked declines in D&A, substantiating patient reports (Table 2). No examples of non-consented care were recorded during the evaluation, compared with incidences as high as 85.1 % from baseline. Additionally, manifestations of non-dignified care such as the woman not being welcomed in a kind and gentle manner, provider not introducing themselves to the patient, and not calling women by their name, were much less frequent during the evaluation phase than at baseline.

**Table 2:** Observed disrespect and abuse

Type of Disrespect and Abuse observed	Baseline *N* = 208 *n* (%)	Evaluation *N* = 459 *n* (%)	Difference between Evaluation and Baseline %
Physical Abuse			
* Fundal pressure applied*	7 (3.4)	1 (0.2)	−3.2
* Lack of anesthesia for episiotomy*	9 (4.3)	0 (0.0)	−4.3
Non-Consented Care			
* Lack of consent for first examination*	177 (85.1)	0 (0.0)	−85.1
* Lack of consent for vaginal examination*	170 (81.7)	0 (0.0)	−81.7
Non-Confidential Care			
* Examination performed in way or setting that other clients could hear*	11 (5.4)	42 (9.2)	3.8
* Health providers discussed mother’s private health information in a way or setting that others could hear*	42 (20.2)	143 (31.2)	11.0
Lack of Privacy			
* No partitions separating beds*	179 (86.1)	118 (25.7)	−60.4
* Partitions did not give privacy/not adequately closed to provide privacy*	76 (36.5)	199 (43.4)	6.9
* Mother not well covered*	133 (63.9)	14 (3.1)	−60.8
Non-Dignified Care			
* Mother not welcomed in a kind and gentle manner*	51 (24.5)	0 (0)	−24.5
* Mother not told where to go in antenatal ward*	28 (13.5)	0 (0)	−13.5
* Delivering service provider did not congratulate the mother after birth*	55 (26.3)	7 (1.5)	−24.8
* Provider did not introduce herself to mother*	194 (93.3)	55 (12.0)	−81.3
* Mother not cleaned after birth and third stage of labor*	33 (26.1)	3 (0.7)	−25.4
* Mother not called her name throughout interactions*	123 (60.0)	1 (0.2)	−59.8
* No bed ready for mother*	113 (54.3)	96 (20.9)	−33.4
* Bed provided to mother was not clean*	135 (65.9)	6 (1.3)	−64.6
* No pad provided to the mother after birth*	17 (8.2)	439 (95.6)	87.4

## Enabling factors for success

We strongly believe that the participatory process and sustained engagement around reducing disrespect and abuse during childbirth contributed to substantial changes in the culture of the study facility, improvement of intermediary outcomes, and, potentially, to remarkable declines in incidence of disrespect and abuse.

In an assessment of the causes and manifestations of disrespect in hospital settings, Leape et al. stated that while the “origins of disrespectful behavior may reside in the personality characteristics of individuals and their responses to stressful environments,” the “expression [of disrespectful behavior] is learned behavior and it thrives in a culture that tolerates and supports disrespect” [9]. Therefore, Leape argues, the elimination of disrespectful behavior requires an organizational culture change. Through the package of discrete interventions and sustained engagement of providers and administrators, the program initiated an open dialogue around the critical issue of D&A and demonstrated that cultural change is possible. The results presented here indicate that important progress towards establishing respectful care as a cultural norm can be achieved in a relatively short period.

Beyond the specific intervention activities, this change in culture was facilitated by the leadership of several key champions within the study facility, including the hospital matron, head of the department of obstetrics and gynecology, the maternity ward matron, and the members of the technical working group. By modeling respectful care, making themselves available to participate in all interventions, and ensuring that dialogue around disrespect and abuse was included in all department meetings, these key staff demonstrated a commitment to respectful maternity care as a core institutional value and were instrumental to the changes observed.

Over the course of the program, our staff observed a notable shift in organizational culture and the pride the facility took in its achievements was palpable. Just as provider burnout, structural abuse, and individual biases may create reinforcing patterns of abuse [10, 11], we believe that empowered women, satisfied providers and patients, and strong communication can create a virtuous reinforcing cycle which fosters individual and institutional commitments to promote and embody respectful care. Additional insight into the enabling factors for the observed successes could come from the women who sought care at the study facility, although this was not included in our study. In particular, qualitative interviews with women who gave birth at the study facility both before and after the intervention could provide rich, detailed information about how and why their experiences differed.

The culture shift documented here is a necessary but insufficient step to ensuring respectful maternity care. In the absence of significant investments in the health system, many structural contributors to disrespect and abuse remained unchanged. Staff perceptions of the sufficiency of human resources and the availability of necessary supplies and medications was not modified from baseline to evaluation. While some observed and reported manifestations of disrespect and abuse, such as non-consented care, were nearly eliminated from baseline to evaluation, others that are directly impacted by structural and human resource deficiencies did not improve over the course of implementation. These included the prompt attention and attendance to patients from providers, privacy during care, and availability of necessary supplies and sufficient clean beds for patients. Thus, while the culture change achieved through this study is a positive shift, ensuring that all women receive dignified and respectful care will require much more, especially a commitment from the entire health system to invest in the improvement of quality of care.

## Conclusions

The results presented here demonstrate that disrespect and abuse is not an intractable problem. By naming D&A and shining a continuous light on the problem, the culture and norms at the facility began to change and the provision of respectful maternity care became an institutional priority. Through a multilevel participatory process, a low-cost package of interventions, and the establishment of champions at the facility, the program affected positive changes in provider job satisfaction, the relationships between providers and patients and providers and administrators, and in women’s satisfaction with care. These interventions are among the first in Tanzania and elsewhere to demonstrate an effect in reducing D&A, and there is widespread interest in scaling them in the program municipality and beyond. We believe that the enabling factors identified in this paper, as well as the specific interventions employed, have the strong potential to reduce disrespect and abuse and promote a culture of respectful care in other large hospitals in the region, however, a larger and more robust evaluation will be needed to determine their true impact and generalizability.
